# Purification and Characterization of a Novel ~18 kDa Antioxidant Protein from *Ginkgo biloba* Seeds

**DOI:** 10.3390/molecules171214778

**Published:** 2012-12-11

**Authors:** Hao Zhou, Xijuan Chen, Chengzhang Wang, Jianzhong Ye, Hongxia Chen

**Affiliations:** 1 Institute of Chemical Industry of Forest Products, CAF, Nanjing 210042, Jiangsu, China; 2 National Engineering Lab for Biomass Chemical Utilization, Nanjing 210042, Jiangsu, China; 3 Key and Open Lab on Forest Chemical Engineering, SFA, Nanjing 210042, Jiangsu, China; 4 Key Lab of Biomass Energy and Material, Nanjing 210042, Jiangsu, China; 5 Institute of New Technology of Forestry, CAF, Beijing 100091, China

**Keywords:** *Ginkgo biloba* seeds, 18 kDa protein molecule, antioxidant activity

## Abstract

*Ginkgo biloba* seeds are widely used as a food and traditional medicine in China. In the present study, a novel antioxidant protein named GBSP was purified from *Ginkgo biloba* seeds. The protein (GBSP) was purified by homogenization of *Ginkgo biloba* seed powder in saline solution, 70% ammonium sulphate precipitation, filtration on a DEAE-Cellulose52 anion exchange column, gel filtration on a Sephadex G-50 column, and preparative chromatography on a C_18_ column using RP-HPLC. GBSP showed an apparent molecular weight of 18 kDa by SDS-PAGE and MALDI-TOF/MS analyses. The amino acid sequence obtained by MALDI-TOF/TOF MS analysis showed GBSP was a novel protein, as no matching protein in was found the database. The protein exhibited significant antioxidant activities against free radicals such as DPPH, ABTS and superoxide anion and showed higher activity than α-tocopherol in a linoleic acid emulsion assay system. Furthermore, GBSP exhibited notable reducing power and a strong chelating effect on Cu^2+^ and Fe^2+^. Therefore, the present study demonstrates, for the first time, that this novel protein from *Ginkgo biloba* seeds is an excellent antioxidant.

## 1. Introduction

Oxidative stress in living organisms can cause excessive production of reactive oxygen species (ROS) which plays a significant role in induction of oxidative damage related diseases such as cancer, ageing, atherosclerosis, diabetes and inflammation. All organisms possess endogenous antioxidants such as superoxide dismutase, catalase and peroxidase, which can cause removal of ROS to protect them from oxidative damage. However, under extreme oxidative stress, the endogenous antioxidants do not provide comprehensive protection from ROS. Then exogenous antioxidant consumption might be helpful to prevent oxidative stress in the body [1,2,3]. Synthetic antioxidants, such as butylated hydroxyanisole (BHA) and butylated hydroxytoluene (BHT), can only be used at low doses, because they may possess side effects and properties toxic to human health [4,5]. Therefore, searching for safer and effective antioxidants from natural plants is of great interest among researchers.

Many substances with antioxidant activity *in vivo* belong to the protein kind. Proteins, having excellent emulsion type characteristics, can play a mediating role in the oil-water interface, and have an important meaning to remove excess free radicals and restrain membrane lipid peroxidation [6]. Recently, proteins from plant sources have been widely reported to possess antioxidant activity, and some such examples include *Terminalia chebula* fruit protein [7], curry leaves protein [8], rice endosperm protein [9], quinoa seed protein [10], sunflower protein [11], chickpea protein [12], soybean protein [13], sundakai seeds protein [14] and *Phyllanthus niruri* protein [15].

*Ginkgo biloba*, one of the oldest species of tree, has existed on the Earth for two hundred million years, and 70% of *Ginkgo biloba* is from China [16]. The seeds of *Ginkgo biloba* are widely used for foods and traditional medicines in China; they are rich in proteins, carbohydrates, vitamin C, riboflavin, and many other nutrients [17]. Research shows that *Ginkgo biloba* seeds have significant antioxidant activity, and this antioxidant activity was mainly related to its protein compounds [18]. At present, studies about purification and preparation of antioxidant proteins from *Ginkgo biloba* seeds are scanty, and only one kind of antioxidant protein named G4b with a molecular mass of 29,247 Da was purified from *Ginkgo biloba* seed albumin to date [19]. As proteins with antioxidant properties have become a topic of great interest for processing and preservation in pharmaceutical/health food industries, it is necessary to search for new antioxidant proteins from *Ginkgo biloba* seeds. In the present study, we isolated a new antioxidant protein (GBSP) from *Ginkgo biloba* seeds; the structural properties of GBSP including molecular weight, amino acid composition and sequence were analysed and the antioxidant properties of GBSP were also evaluated.

## 2. Results and Discussion

### 2.1. Purification of the Antioxidant Protein

**Figure 1:**
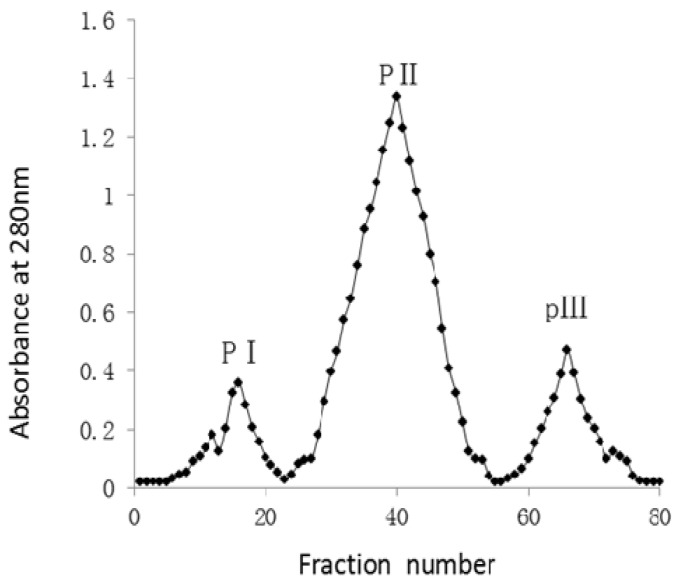
Gel filtration profile of the purified total proteins of *Ginkgo biloba* seeds on a Sephadex G-50 column. Three peaks (PI, PII and PIII) were eluted from the column.

*Ginkgo biloba* seeds were homogenized in 0.14 mol/L NaCl solution dissolved in 0.02 mol/L citrate phosphate buffer, the homogenate was filtered through filter paper and the filtrate was centrifuged, then ammonium sulphate was added to the supernatant to 70% saturation to precipitate proteins. The saline solution extract of *Ginkgo biloba* seeds, showed 51% antioxidant activity by a DPPH radical scavenging assay, and the crude proteins obtained by ammonium sulphate precipitaton showed 61% antioxidant activity. Then the crude proteins dissolved in 0.02 mol/L citrate phosphate buffer were passed through a 3,000 MW polysulfone membrane to remove ammonium salts and other low molecular weight components such as free sugars or polyphenols, which could contribute to the antioxidant activity, and the filtered protein solution was further applied onto a DEAE-Cellulose 52 column. Then the purified total proteins were applied onto a Sephadex G-50 column and eluted with 0.02 mol/L citrate phosphate buffer. Fractionation of the protein resulted in three peaks (PI, PII and PIII) as monitored at 280 nm (Figure 1).

**Figure 2:**
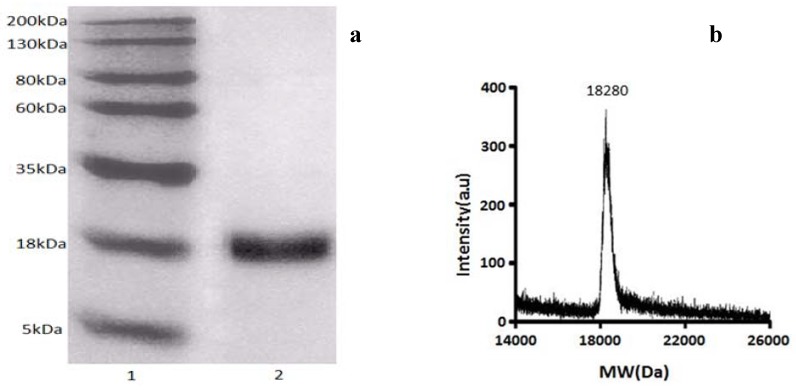

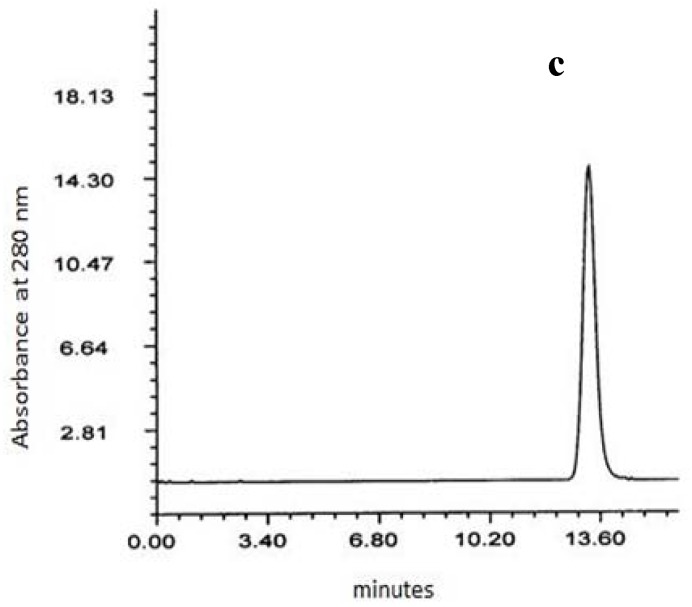
(**a**) SDS-PAGE of purified antioxidant protein from *Ginkgo biloba* seeds. Lane 1, Molecular weight marker; Lane 2, the purified antioxidant protein (GBSP); (**b**) chromatogram of purified protein (GBSP) by MALDI/MS analysis; (**c**) HPLC chromatogram of purified protein (GBSP).

The protein content of the peaks was estimated by the Kjeldahl method. The antioxidant activity of each fraction from the peaks was determined by a DPPH radical scavenging assay. Next the PII protein peak, a symmetrically eluting peak, with comparatively high abundance and the maximum antioxidant activity, was further separated and purified by RP-HPLC on a semi-preparative C_18_ column. One single peak was obtained (Figure 2c).

Table 1 shows the yields and the antioxidant activity of ammonium sulphate precipitated protein, peak I, peak II, peak III and GBPS in comparison with BHA. The antioxidant activity of these protein samples at various concentrations was tested by a DPPH radical scavenging assay, samples were mixed with 2 mL of 0.1 mM DPPH in 95% ethanol, and the absorbance of the resulting solution was read at 517 nm. A lower absorbance represents a higher DPPH scavenging activity. The 70% ammonium sulphate precipitated protein showed 61% antioxidant activity when used at 600 μg/mL concentration. The peak II protein exhibited a highest antioxidant activity of 78% at 50 μg/mL, whereas peak I and peak III protein showed antioxidant activity of 30% and 28% at 400 μg/mL, respectively. Meanwhile, the peak II protein showed a highest yield of 8.11%, whereas peak I and peak III protein showed yields of 1.85% and 2.36%, respectively. The GBSP purified by RP-HPLC showed antioxidant activity of 81% at 50 μg/mL, more efficient than BHA (72 μg/mL) that showed only 65% antioxidant activity.

### 2.2. Characterization of Antioxidant Protein (GBSP) from Ginkgo biloba Seeds

The results of SDS-PAGE electrophoresis of GBSP are shown in Figure 2a. The GBSP showed only one band with an approximate molecular weight of 18 kDa. Through further MALDI/MS analysis, it was shown that GBSP was a protein with exact molecular mass of 18.28 kDa (Figure 2b). The protein purity was further confirmed by reverse phase HPLC that showed single peak (Figure 2c). Thus the above results can indicate homogeneity of the purified protein (GBSP).

### 2.3. Amino Acid Composition

The amino acid composition of the GBSP in the present study is shown in Table 2. GBSP was rich in Glu, Ser, His, Asp, Gly, Ala, Leu and Lys compared with the other amino acids. The total essential amino acid contents of GBSP were 54.22 g/100 g protein, which is higher than the recommended reference values (33.9 g/100 g protein, Table 3). Moreover, the amino acid scores, particularly Ile, Met + Cys, Phe + Tyr, Thr, Trp and His of GBSP were much higher than the recommended FAO/WHO standard (Table 3).

### 2.4. Determination of Peptide Mass Fingerprinting (PMF) and Amino Acid Sequence Analysis

**Figure 3:**
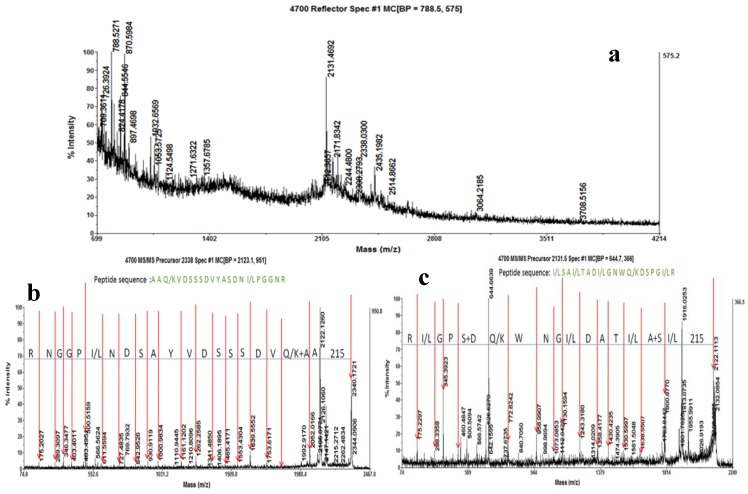
Peptide mass fingerprinting (PMF) and amino acid sequence of GBSP. (**a**) The MALDI-TOF spectrum of GBSP; (**b**) the amino acid sequence of the 2131 Da peptide; (**c**) the amino acid sequence of the 2338 Da peptide.

The PMF of GBSP was acquired by MALDI-TOF-MS. The MALDI-TOF spectrum of GBSP generated from trypsin digestion is shown in Figure 3a. The size of the peptide ions varies from *m/z* 709.36 to 3708.51. The most abundant peptide ions are *m/z* 788.52, 870.59, 2131.46 and 2338.03. Other relatively weak peaks contain *m/z* 844.55, 2171.83, and so on. Analyses by the MASCOT search program suggested that no previously described proteins were found to match GBSP data.

Two peptides of nominal mass 2131Da and 2338Da, with relatively high ionization efficiency, were subjected to MS-MS analysis for sequencing. The amino acid sequence of (a) the 2,131 Da peptide is: I/L S A I/L T A D I/L G N W Q/K D S P G I/L R; (b) the 2,338 Da peptide is: A A Q/K V D S S S D V Y A S D N I/L P G G N R (Figure 3b,c). We failed to identify any proteins in the database by searching with the peptide sequences described above, suggesting that GBSP is a newly discovered protein that has not been extensively characterized previously.

### 2.5. Antioxidant Properties of GBSP

#### 2.5.1. DPPH Radical Scavenging Activity

**Figure 4:**
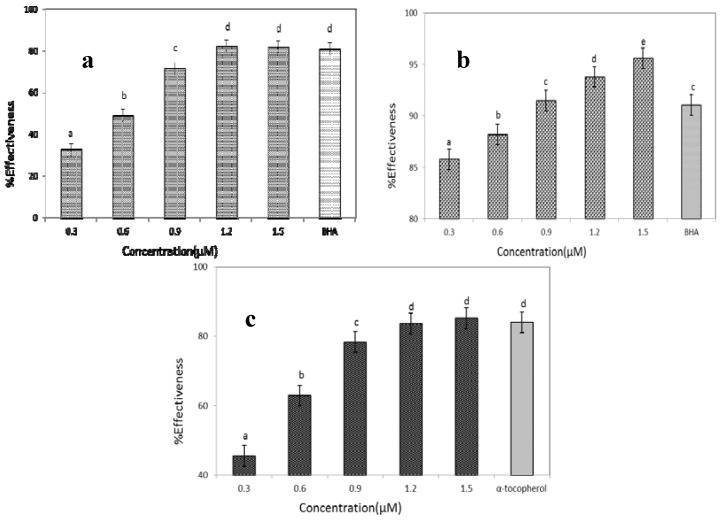
Free radical scavenging activity. (**a**) DPPH radical scavenging activity of GBSP (0.3–1.5 μM) and BHA (400 μM), ^a–d^ means having same superscript are not different (*p* > 0.05); (**b**) ABTS radical scavenging activity of GBSP (0.3–1.5 μM) and BHA (400 μM), ^a–e^ means having same superscript are not different (*p* > 0.05); (**c**) Superoxide anion radical scavenging activity of GBSP (0.3–1.5 μM) and α-tocopherol (400 μM), ^a–d^ means having same superscript are not different (*p* > 0.05). The results are shown as means ± SD (n = 3).

DPPH radical scavenging capacity has been used as primary characterization in antioxidant activity analysis [20]. The DPPH radical-scavenging activity of GBSP increased with the increase in concentration from 0.3 to 1.5 μM. As shown in Figure 4a, GBSP exhibited 45% inhibition at 0.3 μM concentration, and a maximum of 82% inhibition was obtained at 1.2 μM concentration. The scavenging activity remained constant above 1.2 μM concentration. The standard BHA at 400 μM concentration showed DPPH radical scavenging activity of 75%–80%, which indicated the scavenging ability of GBSP was higher than that of standard BHA.

#### 2.5.2. ABTS Radical Scavenging Activity Assay

The ABTS test is widely used for assessing antioxidant activity in foods. A comparison of inhibition of ABTS radical versus concentration of GBSP is shown in Figure 4b. It exhibited a dose-dependent inhibition of the ABTS radical scavenging activity, GBSP showed significant radical scavenging activity of more than 85% inhibition at all concentrations (0.3–1.5 μM), and the scavenging activity of GBSP at concentration from 0.9 to 1.5 μM was higher than the standard BHA (400 μM).

#### 2.5.3. Superoxide Anion Radical-Scavenging Assay

The superoxide anion radical, one of the strongest free radicals in cellular oxidation reactions, is a harmful factor that can induce ageing and destroy cell membranes. And it is also considered to play an important role in the peroxidation of lipid [21]. The scavenging activities of the GBSP on the superoxide anion radical, are shown in Figure 4c. There was a dose-dependent relationship between concentration and antioxidant activity of GBSP. When the concentration of GBSP increased from 0.3 to 1.5μM, the inhibition rate increased rapidly. When concentration of GBSP was 1.5 μM, it reached the maximal inhibition rate of 85.2%. While α-tocopherol provided about 83% inhibition at 400 μM concentration, the inhibitory activity of GBSP was more efficient than that of α-tocopherol.

#### 2.5.4. Inhibition of Linoleic Acid Autooxidation

**Figure 5:**
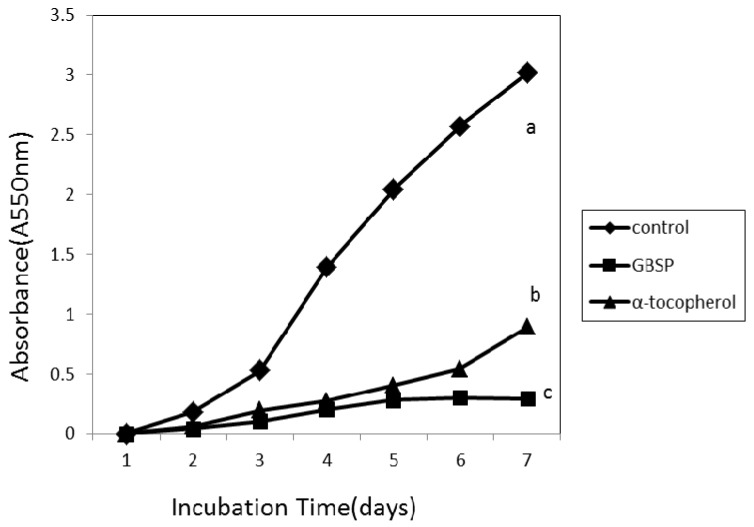
Lipid peroxidation inhibitory activity of GBSP. The activity was measured in a linoleic acid oxidation system for 7 days. α-Tocopherol was used as positive control. ^a–c^ Means having same superscript are not different (*p* > 0.05). The results are shown as means ± SD (n = 3).

When free radicals attack membranes, which are high in lipids, they form lipid peroxides. Peroxidation of lipids is a complex process that involves formation and propagation of lipid radicals. Peroxidation can affect the nutritive value of foods and may cause disease [22]. The inhibitory effect of GBSP on lipid peroxidation was assessed with linoleic acid as lipid phase model system using iron-dependent system as a catalyst. As shown in Figure 5, the GBSP was compared with the natural antioxidant α-tocopherol for a period of seven days. The oxidation of linoleic acid was markedly inhibited by the addition of GBSP, and it was more effective when compared to α-tocopherol. The inhibitory activity of α-tocopherol decreased with time, while GBSP was more stable over time. The inhibitory ratio of GBSP was 90.39% at the seven day, which was higher than that of α-tocopherol (70.53%).

#### 2.5.5. Reducing Power Assay

**Figure 6:**
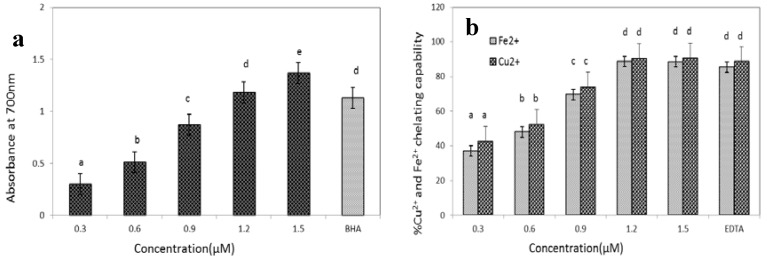
(**a**) Reducing power of GBSP (0.3–1.5 μM) and BHA (400 μM), ^a–e^ means having same superscript are not different (*p* > 0.05); (**b**) Cu^2+^ and Fe^2+^ chelating activity of GBSP (0.3–1.5 μM) and EDTA (60 μM), ^a–d^ means having same superscript are not different (*p* > 0.05). The results are shown as means ± SD (n = 3).

The reducing power assay is often used to evaluate the ability of an antioxidant to donate an electron or hydrogen. Sometimes the reducing power of a compound may serve as an indicator of its potential antioxidant activity [23]. The reducing power of GBSP was measured by transformation of the Fe^3+^ to ferricyanide complex to the ferrous form. As shown in Figure 6a, the reducing power (absorbance at 700 nm) of GBSP of different concentration exhibited a dose-dependent effect, and the maximum absorbance (1.37) was found at 1.5 μM concentration while BHA showed absorbance of 1.13 at 400 μM concentration. The reducing power shown by GBSP is superior to standard BHA.

#### 2.5.6. Metal Ion Chelating Activity

Transition metal ions, such as Cu^2+^ and Fe^2+^, can catalyse the generation of ROS, resulting in lipid peroxidation and DNA damage [24]. Therefore, the chelation of metal ions would retard the lipid peroxidation chain reaction. Figure 6b demonstrates the ability of GBSP to chelate the transition metal ions Cu^2+^ and Fe^2+^, The Cu^2+^ and Fe^2+^ chelating capabilities of GBSP was increased with increased concentration. It showed that GBSP exhibited maximum Cu^2+^ chelating effect of 90% and Fe^2+^ chelating effect of 88% at 1.2 μM, its chelating capabilities were equivalent to EDTA at 60 μM.

### 2.6. Discussion

The *Ginkgo biloba* seed has a relatively high content (10%–15%) of protein, and it has a great economic value in the food industry as a functional additive. However, the study of its active proteins is scanty. Wang and Ng reported isolation of an antifungal protein with a molecular weight of 13 kDa from *Ginkgo biloba* seeds [25]. Although it was reported that albumin and globulin separated from *Ginkgo biloba* seeds possess antioxidant activity [26], other chemical components such as phenols and polysaccharides also have antioxidant activities, so it was uncertain which component was the main contributor to the antioxidant activity. Later, Huang and Deng reported an antioxidant protein with a molecular weight of 29 kDa obtained from albumin of *Ginkgo biloba* seeds [19]. This obtained protein has a higher molecular weight, and so far, there are no reports on antioxidant proteins with smaller molecular weight obtained from albumin of *Ginkgo biloba* seeds.

The present study was carried out to isolate and characterize an active antioxidant protein (18 kDa) obtained from the saline solution extract of *Ginkgo biloba* seeds. The saline solution extract showed antioxidant activity using the DPPH radical scavenging assay, but because it contained other chemical components such as polyphenols and polysaccharides, which were reported to have antioxidant activities [27], therefore, the components of polyphenols and polysaccharides were removed by polysulfone membrane and DEAE-Cellulose 52 separation. Then by Sephadex G-50 column and semi-preparative C_18_ column chromatography, we isolated and purified the protein GBSP to homogeneity. SDS-PAGE and MALDI-TOF-MS analysis of the protein demonstrated an approximate molecular weight of 18.28 kDa.

The partial amino acid sequences of the protein GBSP were obtained from two prominent peptide fragments by MALDI-TOF-MS analyses, and the compositions of amino acids in GBSP were determined by a Biochrom 30+ Amino Acid Analyzer. It showed that GBSP contained abundant His, Ile, Ser, Leu, Gly, Asp, Ala, Trp, Cys, Glu, Met and Phe amino acids. Both composition and sequences of amino acids play a role in antioxidative properties of protein [28]. It has been reported that aromatic amino acids such as Tyr, Phe and Trp and the sulfur-containing amino acid, Cys, possess high free radical scavenging activity [29]; it is commonly believed that His, Met, Leu, Ala and Cys have free radical-scavenging and iron-binding activities due to their special structure of characteristics [22]; in addition, the basic amino acids such as Lys and Arg, and the acidic amino acids, such as Asp and Glu, exercise antioxidant activity by chelating metal ions [30]. So, presence of His, Asp, Ala, Trp, Cys, Glu, Met and Phe amino acids in the peptide fragments of GBSP, can contribute to the antioxidant potential of the protein (GBSP).

Oxidative stress inducers could cause the formation of reactive oxygen species (ROS) and free radicals. Free radicals contain unpaired electrons which react rapidly with other groups in the biosystem and could cause enhanced lipid peroxidation and cell injury [31]. The experimental results indicate that the GBSP effectively scavenges free radicals such as DPPH, ABTS and superoxide anion radicals at the lower concentrations than the standards BHA and α-tocopherol. It is presumed that GBSP may well act as an electron donor and can react with free radicals to convert them to more stable products and terminate radical chain reactions. Thus GBSP could be an effective scavenger of free radicals.

Along with free radical scavenging activity, the antioxidant activity of GBSP was further confirmed by lipid peroxidation inhibition assay in linoleic acid model system. Lipid peroxidation of the linoleic acid model system is thought to proceed via radical-mediated abstraction of hydrogen atoms from methylene carbons in polyunsaturated fatty acids [22]. The experimental results indicate that GBSP markedly inhibited the oxidation of linoleic acid, and it is more effective than α-tocopherol. This was presumed to be because GBSP react with radicals in the system and inhibit the propagation cycle of lipid peroxidation. So, the GBSP is effective inhibitor of lipid peroxidation.

It has been shown that the antioxidant effect increases exponentially as a function of the development of the reducing power, so samples with higher reducing power have better abilities to donate electrons or hydrogens [32]. The GBSP exhibited maximum reducing power at lesser concentration compared to BHA. This suggests that GBSP was a potent antioxidant, which agrees with the radical scavenging activity.

Transition metal ions, such as Cu^2+^ and Fe^2+^, are essential mineral for normal physiology, but excess can result in lipid peroxidation and DNA damage. If they undergo the Fenton reaction, these reduced metals can catalyse the generation of reactive oxygen species such as the hydroxyl radical and superoxide anion [33]. Therefore, the chelation of transition metal ions would retard the oxidation reaction. The antioxidant factors of the protein GBSP were found to be capable of binding Cu^2+^ and Fe^2+^ ions, the GBSP exhibited equivalent chelating capabilities compared to EDTA. These results indicate that GBSP could exert a protective effect under conditions of the Fenton reaction.

## 3. Experimental

### 3.1. Plant Material

*Ginkgo biloba* seeds were obtained from the Taixin Market of Jiangsu province and identified to be Longyan species by Qinan Wu, a taxonomist from Nanjing University of Traditional Chinese Medicine. The seeds were shelled, husked and then stored in the refrigerator at −5 °C until used.

### 3.2. Chemicals/Materials

Ammonium sulphate, α-cyano-4-hydroxycinnamic acid, bovine serum albumin (BSA), trypsin, α-tocopherol, trichloroacetic acid, ammonium persulfate and amino acid standards were purchased from Sigma Chemical Co. (St. Louis, MO, USA). 1,1-Diphenyl-2-picrylhydrazyl (DPPH), butylated hydroxyanisole (BHA), linoleic Acid, and ethylene diaminetetraacetic acid (EDTA) were procured from Nanjing Jitian Chemical Reagents Company, Nanjing, China. Ultrafiltration device (Pall, New York, NY, USA), DEAE-Cellulose52 (Whatman, Maidstone, UK), Sephadex G-50 (Pharmacia, Uppsala, Sweden), Centrifuge (Thermo, Boston, MA, USA), Freeze Drier (Virtis, New York, NY, USA), Analytical, Preparative HPLC (shimadzu, Kyoto, Japan) and MALDI-TOF/MS (ABI, Carlsbad, CA, USA) were used for centrifugation, lyophilization, purification, MS and amino acid sequence analysis, respectively.

### 3.3. Purification of the Protein

All the purification operations were done at a temperature from 2 °C to 5 °C. *Ginkgo biloba* seeds (500 g) were freeze-dried, crushed and then defatted by Soxhlet extraction with *n*-hexane at 4 °C for 10 h. The defatted flour was air-dried, then homogenized in 7 L of 0.14 mol/L NaCl (dissolved in 0.02 mol/L citrate phosphate buffer, pH 7.5). The homogenate was filtered through filter paper and the filtrate was centrifuged at 8,000 rpm at 4 °C for 20 min, then to the supernatant was added ammonium sulphate to 70% saturation to precipitate proteins. After stirring overnight, the crude proteins were collected by centrifugation at 8,000 rpm for 30 min. Crude proteins (50 g) were dissolved in 500 mL 0.02 mol/L citrate phosphate buffer and filtered through a 3,000 MW polysulfone membrane. The filtered solution was loaded on DEAE-Cellulose 52 column (6 × 80 cm), equilibrated with 0.02 mol/L citrate phosphate buffer (pH 7.5) and eluted with 0.4 mol/L NaCl (dissolved in 0.02 mol/L citrate phosphate buffer, pH 7.5). The fractions were collected at 280 nm using a Buchi C-630 UV detector. The pooled fractions were mixed and lyophilized up to 100 mL. This was loaded on a Sephadex G-50 column (3 × 50 cm), equilibrated with 0.02 mol/L citrate phosphate buffer (pH 7.5) and eluted with the same solution, fractions were collected and monitored at 280 nm. Three peaks, peak I, II, III, were obtained. Each peak fraction was pooled separately and lyophilized. The scavenging activities of peak I, II, III on DPPH free radicals were measured. The peak II with the highest antioxidant activities was further purified by semi-preparative HPLC using a C_18_ column (10.0 mm × 250 mm, 5 μm particles). The column was eluted with water-acetonitrile (95:5) containing 0.1% trifluoroacetic acid (TFA) at a flow rate of 2.0 mL/min. The eluates were detected at 280 nm. The protein obtained by this process was designated as GBSP.

### 3.4. Test for Purity and Molecular Weight of GBSP

The purity and molecular weight of GBSP were assessed according to the method of Secknus [34]. SDS-PAGE was performed with 15% resolving and 5% stacking gels. A set of molecular weight marker proteins (5–200 kDa) were electrophoresed to roughly estimate molecular weight of the proteins.

The purity of GBSP was analyzed by reverse phase HPLC using a C_18_ column (4.6 mm × 250 mm, 5 μm particles). The column was eluted with water-acetonitrile (95:5) containing 0.1% trifluoroacetic acid (TFA) at a flow rate of 1.0 mL/min. The detection was at 280 nm.

The accurate molecular weight of GBSP was determinated by MALDI/MS with the following parameters: nitrogen laser; wavelength of 355 nm, spectrum range of 10–100 kDa; delayed ion extraction in linear mode; accelerating voltage of 20 kV; low mass ion gate was set at 1,500 Da. The instrument was calibrated with bovine serum albumin.

### 3.5. Peptide Mass Fingerprinting (PMF) Analysis and the Amino Acid Sequence Determination of GBSP

PMF and amino acid sequence of GBSP was analyzed by MALDI-TOF/TOF MS as described previously [35]. The sample was first digested with trypsin and incubated at 37 °C overnight. The digestion mixture was dissolved in a solution of distilled water/acetonitrile/formic acid (45:50:5), then the solution was centrifuged, the supernatant was dried and dissolved with 50% acetonitrile solution containing 0.1% TFA and 5 mg/mL α-cyano-4-hydroxycinnamic acid, then it was analyzed by MALDI-TOF/TOF MS with following parameters: positive ion pattern; Nd:YAG laser; wavelength of 355 nm; TOF acceleration potential of 9.1 kV; the grid potential of 6.7 kV. The mass spectrum was analyzed on an ABI 4700 Proteomics Analyser (Applied Biosystems, Foster City, CA, USA) using both MS and MS/MS operating modes. PMF was determined with GPS Explorer software (Applied Biosystems) using the Mascot search algorithm; the peptide sequencing was performed by manual calculation according to MS/MS data.

### 3.6. Protein Estimation and Amino Acid Composition

The protein content at various stages of purification was estimated by the Kjeldahl method [36]. Bovine serum albumin (BSA) was used as the working standard. The amino acids compositions were determined by a Biochrom 30+ Amino Acid Analyzer (Biochrom, Cambridge, UK). The samples were hydrolysed with 6 mol/L HCl for 22 h at 110 °C in a sealed tube filled with nitrogen gas, and then injected for analysis. The amino acid was expressed as g/100 g protein. The amino acid score for the essential amino acids was calculated following the equation:

%Amino acid score = [A/A_0_] × 100

where A = Amount of amino acid per sample protein (g/100g); A_0_ = Amount of amino acid in recommended FAO/WHO (1991) standard protein (g/100 g).

### 3.7. Determination of Antioxidant Activity

#### 3.7.1. DPPH Radical-Scavenging Activity

DPPH radical scavenging activity was measured according to the method of Bersuder [37]. Samples at various concentrations ranging from 0.3 to 1.5 μM dissolved in distilled water were mixed with 2 mL of 0.1 mM DPPH in 95% ethanol. The mixtures were shaken immediately and left for 60 min in the dark at room temperature, and the absorbance of the resulting solution was read at 517 nm. A lower absorbance represents a higher DPPH scavenging activity. The control was conducted in the same manner, except that distilled water was used instead of sample. BHA was used as a standard. The inhibitory percentage of DPPH was calculated as per the formula:

% Scavenging effect = [(A_0_ − A)/A_0_] × 100

where A_0_ = absorbance of control; A = absorbance of sample.

#### 3.7.2. ABTS Radical Scavenging Activity Assay

ABTS radical scavenging activity assay was performed according to Arnao [38] with some modifications. The ABTS stock solution was prepared by mixing ABTS solution (7 mM, 3 mL) and ammonium persulphate (2.45 mM, 15 mL) in distilled water. The mixture was left in the dark at room temperature for 16 h before use. Fresh ABTS working solution was prepared by mixing ABTS stock solution in 0.2 M sodium phosphate buffered saline (pH 7.4) to an absorbance of 0.7 ± 0.02 at 734 nm. Then 50 μL of the sample (0.3–1.5 μM) was added to 5 mL of fresh ABTS working solution. The reaction mixture was kept in the dark for 6 min and absorbance was monitored at 734 nm. BHA was used as a standard. The control was conducted in the same manner, except that distilled water was used instead of sample. The percentage inhibition was calculated using the formula:

% Inhibition = [(A_0_ − A)/A_0_] × 100

where A_0_ = absorbance of control; A = absorbance of sample.

#### 3.7.3. Superoxide Anion Radical-Scavenging Assay

The superoxide anion radical-scavenging ability of GBSP was assessed by the method described by Li [39] with slight modifications. A pyrogallol solution (3 mM) was added to a tube containing a sample that had been dissolved in a Tris-HCl-EDTA buffer (0.1 M, pH 8.2). The mixture was incubated at 25 °C for 10 min and then the optical density (OD) of the solution at 320 nm was measured using a spectrophotometer. α-Tocopherol (400 μM) was used as a standard. The control was conducted in the same manner, except that distilled water was used instead of sample. The antioxidant activity was determined as the percentage of inhibiting pyrogallol autooxidation, using the formula:

% Inhibition = [(A_0_ − A)/A_0_] × 100

where, A_0_ = absorbance of control; A = absorbance of sample.

#### 3.7.4. Inhibition of Linoleic Acid Autooxidation

The antioxidant activity of GBSP was measured in a linoleic acid model system according to the methods of Osawa [40] with some modifications. Sample (3 mg) was dissolved in 50 mM phosphate buffer (pH 7.0, 10 mL) and added to a solution of linoleic acid (0.13 mL) and 99.5% ethanol (10 mL). The total volume was then adjusted to 25 mL with distilled water. The mixture was incubated in a conical flask with a screw cap at 40 °C for 7 days in a dark room. The degree of oxidation of linoleic acid was measured using the ferric thiocyanate method of Mitsuda [41]. The reaction solution (100 mL) incubated in the linoleic acid model system described herein was mixed with 75% ethanol (4.7 mL), 30% ammonium thiocyanate (0.1 mL), and 0.02 M ferrous chloride solution in 3.5% HCl (0.1 mL). After 3 min, the degree of colour development that represents linoleic acid oxidation was measured. α-Tocopherol was used as reference and the distilled water was used as the positive control.

#### 3.7.5. Reducing Power Assay

The reducing power of GBSP was determined according to the method of Yildirim [42]. The sample was dissolved in distilled water to obtain a concentration ranging from 0.3 to 1.5 μM. An aliquot (2.0 mL) was mixed with 0.2 M phosphate buffer (pH 6.6, 2.0 mL) and 1% (w/v) potassium ferricyanide (2.5 mL). The mixtures were incubated at 50 °C for 30 min. Then, 10% trichloroacetic acid (2.5 mL) was added and the reaction mixtures were centrifuged at 3,000 rpm for 10 min. Finally, the supernatant (2.5 mL) was collected and mixed with distilled water (2.5 mL) and 0.1% (w/v) ferric chloride solution (0.5 mL). After a 10 min reaction time, the absorbance was measured at 700 nm. BHA was used as a standard. The distilled water instead of the sample was used as the positive control. Higher absorbance of the reaction mixture indicated higher reducing power.

#### 3.7.6. Metal Ion Chelating Activity

The Cu^2+^ and Fe^2+^ chelating activities of GBSP were determined according to the method of Kong [43] with with minor modification. In the Cu^2+^ chelation assay, 2 mM CuSO_4_ (1 mL) was mixed with pyridine (10%, pH 7.0, 1 mL) and 0.1% pyrocatechol violet (20 μL). After the addition of sample (1 mL), the disappearance of blue color was recorded by measuring the absorbance at 632 nm. In the Fe^2+^ chelation assay, 2 mM ferrous chloride (0.1 mL) was mixed with 0.5 mM ferrozine (1 mL). After the addition of sample (1 mL), the absorbance change was measured at 562 nm. EDTA was used as a standard. An equivalent volume of distilled water instead of the sample was used as the control. The metal ion chelating activity exhibited by the sample was calculated by the following formula:

% Inhibition = [(A_0_ − A)/A_0_] × 100

where A_0_ = absorbance of control; A = absorbance of sample.

### 3.8. Statistical Analysis

All the tests were done in triplicate and data were averaged. Statistical analysis was done in IBM SPSS Statistics 19.0 software (SPSS Inc., Chicago, IL, USA) using one sided Student’s *t*-test. *p* value < 0.05 was considered as statistically significant as comparing to relevant controls.

## 4. Conclusions

In conclusion, a 18 kDa protein molecule isolated from *Ginkgo biloba* seeds was an effective free radical scavenger and transition metal ion chelator, and it can efficiently inhibit lipid peroxidation. The mechanism of its antioxidant action could probably involve quenching of reactive oxygen species, thereby reducing the potential of prooxidants to attack cellular components. These results suggest that the GBSP may serve as a good antioxidant protein for nutraceutical and pharmaceutical applications. However, further studies are required to investigate the *in vivo* antioxidant activities and cytotoxicity of GBSP.

## Figures and Tables

**Table 1:** Summary of purification antioxidant protein from *Ginkgo biloba* seeds.

**Purification steps**	**Total protein ^a^ (g)**	**Yield (%)**	**Inhibition of DPPH assay**	
			Protein concentration (μg/mL)	% Inhibition
1. saline solution extract	50.56 ± 0.43	100	1000	51 ± 4 *
2. 70% (NH_4_)_2_SO_4_ precipitation	26.18 ± 0.27	51.77 ± 0.53	600	61 ± 3 *
3. Gel filtration on Sephadex G-50				
Peak I	0.936 ± 0.040	1.85 ± 0.08	400	30 ± 2
Peak II	4.10 ± 0.04	8.11 ± 0.09	50	78 ± 3 **
Peak III	1.19 ± 0.07	2.36 ± 0.14	400	28 ± 2
4. purification by RP-HPLC on the semi-preparative C18 column				
GBSP	0.650 ± 0.032	1.29 ± 0.06	50	81 ± 3 **
Standard antioxidant			BHA (72 μg/mL)	63 ± 2 **

Antioxidant activity of protein from *Ginkgo biloba* seeds at the concentrations ranging from 50–1,000 μg/mL was determined using DPPH assay, as described in methods. The results are shown as means ± SD (n = 3). *^a^* Data refer to the protein obtained during various stages of purification from 500 g of Ginkgo biloba seeds. * *p* < 0.05 *vs*. control; ** *p* < 0.01 *vs*. control.

**Table 2:** Amino acid composition of purified protein (GBSP) powder (g/100 g protein).

Amino acid	GBSP
aspartic acid (Asp)	10.62
glutamic acid (Glu)	9.33
serine (Ser)	8.55
histidine (His)	11.25
glycine (Gly)	8.72
threonine (Thr)	4.88
arginine (Arg)	1.82
alanine (Ala)	5.37
tyrosine (Tyr)	3.48
cysteine (Cys) valine (Val)	2.02 4.43
methione (Met)	2.05
phenylalanine (Phe)	4.72
isoleucine (Ile)	4.39
leucine (Leu)	7.46
lysine (Lys)	7.16
proline (Pro)	1.37
tryptophan (Trp)	2.38

**Table 3:** Amino acids score (%) of purified protein (GBSP) powder.

Amino acid	GBSP (g/100 g protein)	Standard FAO/WHO (1991) (g/100 g protein)	Score (%)
Ile	4.39	2.8	157
Leu	7.46	6.6	113
Lys	7.16	5.8	123
Met + Cys	4.07	2.5	163
Phe + Tyr	8.20	6.3	130
Thr	4.88	3.4	144
Trp	2.38	1.1	216
Val	4.43	3.5	127
His	11.25	1.9	592
Total	54.22	33.9	
